# Relevance of Group Training for Psychiatrists: an Experiential-Strategic model.

**DOI:** 10.1192/j.eurpsy.2023.2384

**Published:** 2023-07-19

**Authors:** M. Battuello, A. Flore, T. I. Mele, C. L. Zagaria

**Affiliations:** 1 University of Rome Sapienza, Faculty of Medicine and Psychology; 2 IIRIS Integrated Institute for Research and Strategic Intervention, Rome, Italy

## Abstract

**Introduction:**

There are many stressors that lead to burn out and decrease the quality of life of health professionals as a whole and it occurs also to trainee psychiatrists.

Training programs rarely include specific interest in the personal self of students even if they begin to deal with severe human suffering.

Authors present a model of experiential group training in psychiatry that is centred on the person/trainee at the very most.

**Objectives:**

The aim is to focus on unsolved emotional needs of students to allow them to achieve the capacity of relationship with patients. It is not a mere application of empathy but a truth overcoming, for trainees, of major risks of collusion due to reflection of individual conflicts into the patients and/or due to the encounter with strong emotion during clinical training.

**Methods:**

The model is Experiential because it is the space for personal expression and it is Strategic because it is born inside the strategic group training in psychotherapy (Battuello *et al*. Psichiatria e Psicoterapia 2022; 41, 2, 65-82).

The conductor of the group carries on her/his skin the experience of own training group, to be brought into the trainees’ one.

This is an original approach because the epistemology of the model came directly from the experience.

The group is led by a psychotherapist that plays an active part inside the process instead of being only a facilitator.

The main focus is to allow students to express themselves that includes various steps such as: tuning with their own experiences/emotions, freedom of expressing them to the group, active listening to the other and response to the same other even when feelings don’t resonate but instead are divergent.

This phase is related to the conductor’s participation that is totally immersed into the group bringing personal feelings, stories and emotions to create an undifferentiated space, free from hierarchical roles.

During a second period, students can access a more mature relational capacity that carries the group to a phase of individuation of the self that also engages professional issues.

**Results:**

Students in the group question themselves: it is the root of every health professional that has to explore and overcome personal relational issues. Only after the expressiveness phase, as authors name this part of the training, an individuation phase is truly possible that leads to the definition of the professional.

**Conclusions:**

The training group is necessary for students to explore the wider range of emotions, expressing personal ones, accepting others’, experiencing the tolerance to their frustration, and emerging as professional, that is, in few words, professional of the relationship, the key and the basement of the psychiatrist.

The training in mental health should include, at first, the taking care of the person/student as it is proposed by authors inside the group model.

**Disclosure of Interest:**

None Declared

